# Supplementary material for: Structure–Activity Relationship of the Dimeric and Oligomeric Forms of a Cytotoxic Biotherapeutic Based on Diphtheria Toxin
Source: Biomolecules. 2022 Aug 12;12(8):1111. doi: 10.3390/biom12081111 (PMC9406121; doi:10.3390/biom12081111)
Supplement: Supplementary file 1 [file biomolecules-12-01111-s001.zip › biomolecules-1830840-supplementary.pdf]

Supplementary Materials

1. Brief description of SVD-EFA method

The set of singular values (SV) and singular vectors approximate the data set (**Eq. 2**), where **A** is a matrix whose columns represent data, **U** and **T** are unitary matrices representing singular vectors, and **S** is a diagonal matrix whose entries are SV.

$$\mathbf{A} = \mathbf{USV}^{\mathrm{T}}$$

The columns of **U** correspond to basis states in which linear combinations form each $I(q)$ profile in **A**, while the column of **V** describes the contribution of each basis state for a given scattering profile. Here, the data matrix comprises subtracted SAXS profiles arranged in a time-depended way. Many highly correlated SV indicates the number of different scatterers in the solution. SVD combined with EFA allows identifying ranges within an elution peak where separate species elute via monitoring rapid changes in the number of significant SV as $I(q)$ profiles are added or removed from the matrix **A**. When all peak ranges are identified, the singular vectors in **V** can be rotated and converted into physically meaningful elution profiles.

Table S1. The binding energy score between two N-terminal domains of DT and IL-13 (or C-terminal domain for DT), and the hydrodynamic parameters for the generated fusion cytotoxin models and the Diphtheria toxin model calculated with the HullRad algorithm (http://52.14.70.9/index.html).

	DT	Model 1	Model 2	Model 3	Model 4	Model 5
Binding energy, mean±SD (kcal•mol^{-1})	2489±24	1194±40	1066±79	1448±44	1465±34	1124±23
Molecular mass (g•mol^{-1})	58480	55053	55053	55053	55053	55053
Partial specific volume $\tilde{V}$ (ml•g^{-1})	0.734	0.736	0.736	0.736	0.736	0.736
Anhydrous volume sphere radius R_0 (Å)	25.72	25.23	25.23	25.23	25.23	25.23
Anhydrous radius of gyration Rg (Å)	25.43	28.09	26.18	27.20	26.73	26.71
Maximum dimension Dmax (Å)	85.20	85.57	85.21	85.21	85.21	85.20
Axial ratio (a/b)	1.54	1.42	1.47	1.49	1.51	1.49
Frictional ratio (Rt/R_0) (f/f_0)	1.27	1.37	1.33	1.33	1.32	1.32
Translational diffusion coefficient Dt (µm^2•s^{-1})	65.5	62.1	63.8	63.9	64.5	64.2
Translational hydrodynamic radius Rt (Å)	32.75	34.50	33.59	33.53	33.23	33.38
Sedimentation coefficient (from Rt) S (s)	4.19e-13	3.72e-13	3.82e-13	3.83e-13	3.86e-13	3.84e-13
Rotational diffusion coefficient Dr (s^{-1})	3.76e+06	3.31e+06	3.55e+06	3.55e+06	3.63e+06	3.60e+06
Rotational hydrodynamic radius Rr (Å)	34.96	36.47	35.63	35.64	35.36	35.48
Rotational correlation time τc (from Rr) (ns)	44.31	50.28	46.91	46.92	45.85	46.31

Table S2. Non-linear regression parameters for the ELISA with IL13R 2 receptor and the cytotoxin monomer, dimer, and oligomers. Two models were used, sigmoidal to derive EC_{50} and quadratic to derive apparent K_d values. Fit quality was evaluated by the R square values, parameter standard errors, and 95% confidence bands.

			Monomer	Dimer	Oligomers
FIT SUMMARY	Sigmoidal	EC50 (nM)	1.877	6.404	6.834
		SE	0.289	0.728	1.034
		95% CI	1.394 to 2.771	5.004 to 8.542	5.028 to 9.711
		R^2 (weighted)	0.9759	0.9945	0.9950
		Adjusted R^2 (weighted)	0.9679	0.9926	0.9934
		EC50 ratio ± SE	1.0	3.4±0.7	3.6±0.8
	Quadratic	Kd (nM)	0.06744	4.888	4.410
		SE	0.3183	0.6992	0.6229
		95% CI	??? to 1.056	3.500 to 6.608	3.108 to 5.893
		R^2 (weighted)	0.9736	0.9943	0.9948
		Adjusted R^2 (weighted)	0.9683	0.9932	0.9937
		Kd ratio ± SE	1.0	72±342	65±309

??? - means that the lower limit of the confidence interval could not be determined

Table S3. SAXS-derived parameters for selected datasets (**ESI**). Datasets highlighted in bold were selected for further analysis.

Dataset	R_g [Å]		Molecular weight [kDa]				D_{max} [Å]	
	Guinier	BIFT	Bayes	V_c	V_p	S&S; shape	BIFT	S&S
1.0	48.0±0.4	54.9±1.1	94.2	82.8	113.3	nd; random-chain	263.5±12.5	nd
1.1	105.2±1.1	135.0±9.0	964.9	2810.0	3870.0	1050.0; compact	413.0±33.4	353.0
1.2	54.1±0.4	92.3±6.3	185.8	171.5	209.2	198.3; extended	360.4±24.8	196.0
1.3	40.4±0.1	46.4±1.0	85.7	77.8	95.6	89.8; flat	280.5±16.5	144.0
**1.3T**	**40.4±0.1**	**43.3±0.0**	**85.7**	**77.8**	**95.6**	**90.4; flat**	**168.9±6.9**	**145.0**
**1.4**	**60.5±0.2**	**66.9±0.6**	**242.6**	**238.0**	**295.1**	**234.4; flat**	**214.2±9.3**	**206.0**
1.5	233.0±7.0	336±6.5	nd	20800.0	22500.0	513.6; compact	1100.0±57.9	477.0
**2.0**	**48.3±0.3**	**51.3±0.4**	**169.6**	**154.6**	**187.2**	**173.1; flat**	**178.6±9.5**	**175.0**
3.0	96.2±0.8	144.0±3.8	873.0	934.8	1000.0	880.1; extended	670.8±31.4	350.0
3.1	149.0±1.0	132.0±0	nd	3300.0	3200.0	627.9; extended	408.5±0.0	486.0
**3.2**	**65.2±0.5**	**70.0±0.1**	**790**	**967.1**	**948.3**	**nd; nd**	**186.6±6.8**	**nd**
3.3	210.0±1.6	236.0±3.3	nd	1900.0	2200.0	nd; random-chain	917.3±21.6	nd
3.4	92.6±0.3	142.0±0.0	318.4	339.5	391.6	nd; random chain	403.4±0.2	nd
4.1	243.0±0.5	263.0±0.1	nd	1800.0	1300.0	513.1; flat	981.7±2.5	477.0
4.2	172.0±0.4	141.0±0.0	nd	5500	5000.0	482.3; extended	400.0±0.1	475.0
**4.3**	**108.0±0.5**	**103±0.0**	**873.0**	**1200.0**	**1200.0**	**650.1; extended**	**402.0±0.2**	**385.0**
4.4	310.0±2.0	332.0±0.4	nd	10000.0	7600.0	nd; random-chain	1100.0±2.3	nd
**5.1**	**35.2±0.2**	**37.2±0.4**	**80.8**	**75.6**	**89.8**	**82.0; flat**	**124.6±7.5**	**127.0**
5.2	56.8±0.2	45.7±0.2	138.2	122.6	172.1	174.2 flat	171.7±7.1	203.0
5.3	245.0±2.3	157.0±0	nd	4500.0	4000.0	nd; flat	400.0±0	nd

nd – not determined

Table S4. Additional parameters calculated for selected datasets.

Dataset	d	V_P [Å^3×10^6]	ρ [g × cm^{-3}]	Flexibility[a]	Shape (Porod)[b]	Shape ($P(r)$)[c]	Aggregation
1.3	1.9	0.48	0.34	Y	extended chain	flexible, aggregation	subtle
1.3T	2.0	0.42	0.40	Y	extended chain	flexible linker[d]	no
1.4	2.4	0.93	0.57	Y	extended chain	flexible,	subtle
2.0	2.0	0.92	0.36	Y	extended chain	multidomain	no
3.2	4.8	2.10	0.77	N	hard sphere	flexible linker	subtle
4.3	4.0	3.11	0.65	N	hard sphere	globular, ensemble[e]	moderate
5.1	1.5	0.70	0.22	Y	extended chain	ensemble, aggregation flexible linker	no

[a]flexibility based on plateau on PD plot and shape of dimensionless Kratky plot, [b]shape estimated on value of Porod exponent, [c]shape estimated from P(r), [d]two domains connected via linker, [e] set of different molecules in the solution

Table S5. DENNS models characterization.

Parameter	1.3T	1.4	2.0	3.2	4.3	5.1
Mean RSC[a]	0.931	0.744	0.798	0.756	0.865	0.806
StDev RSC	0.021	0.050	0.044	0.019	0.020	0.034
Aligned reconstructions	19	20	39	97	96	94
Correlation score	0.991	0.952	0.987	0.991	0.993	0.991
FSC[b] Resolution [Å]	36.3	62.4	50.5	102.6	104.8	39.8
Refinement steps	10078	4800	10999	10999	10999	5521
Final χ^2	0.113	0.142	0.082	0.014	5.58	0.008
R_g [Å]	43.4	69.5	54.7	74.8	102.2	39.28
V [Å^3×10^6]	0.32	1.05	0.60	2.78	5.27	0.26

[a]Real Space Correlation [b]Fourier Shell Correlation.

Table S6. Characterization of conformational ensembles and oligomers for selected datasets[a]

Dataset	Method	Ensemble size or oligomeric state	Conformer percentages [%] and name[b]	R_g [c] [Å]	D_{max} [c] [Å]
1.3T	BILBOMD	1	100.0 (*b1-1*)	34.5	111.2
		2	72.0 (*b2-1*), 28 (*b2-2*)	34.2	107.3
		3	44.6 (*b3-1*), 17.7 (*b3-2*), 37.7 (*b3-3*)	34.3	108.1
	MultiFoXS	1	100.0 (*e1-1*)	34.8	106.8
		2	76.5 (*e2-1*), 23.5 (*e2-2*)	35.6	120.5
		3	17.9 (*e3-1*), 63.8 (*e3-2*), 18.3 (*e3-3*)	35.5	119.7
		4	23.8 (*e4-1*), 36.4 (*e4-2*), 33.8 (*e4-3*), 5.9 (*e4-4*)	35.2	113.5
	SASREF	1	100.0	34.7	105.3
		1	100.0	35.2	106.9
		1	100.0	35.4	106.9
		1	100.0	35.3	109.5
		1	100.0	34.2	105.0
1.4	FoXSDock, manual docking	trimer	nd. (*t-1*)	58.1	188.3
		trimer	nd. (*t-2*)	61.2	210.9
2.0	FoXSDock	dimer	nd. (*e2-1-e2-2*)	48.2	151.7
		dimer	nd. (*e4-3-e4-3*)	49.2	170.7
5.1	BILBOMB	1	100.0	34.0	112.9
	MultiFoXS	1	100.0	35.0	110.8
	SASREF	1	100.0	35.3	117.3
		1	100.0	34.7	113.2

		1	100.0	34.8	115.7
		1	100.0	34.6	117.9
		1	100.0	35.2	116.9
		1	100.0	34.8	117.9
		1	100.0	34.7	115.5
		1	100.0	34.3	115.0
		1	100.0	34.5	113.0
		1	100.0	34.5	116.6

[a]for each ensemble sizes the highest scoring was selected (by a given method) [b]some conformers generated by MultiFoXS have the same structures *e2-1* = *e3-2*, *e2-2* = *e3-3* = *e4-4*, *e1-1* = *e4-1* [c]determined from $P(r)$ function.

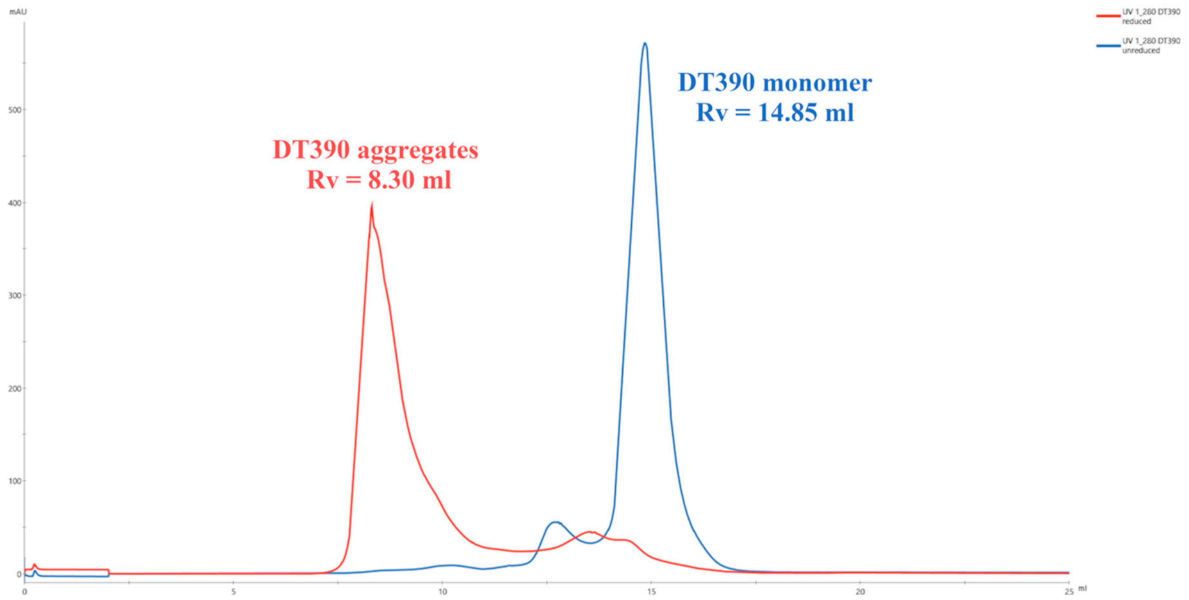

Figure S1. TCEP reduction of DT390 protein. DT390 monomer (blue) and DT390 aggregates formed upon reduction by TCEP (red).

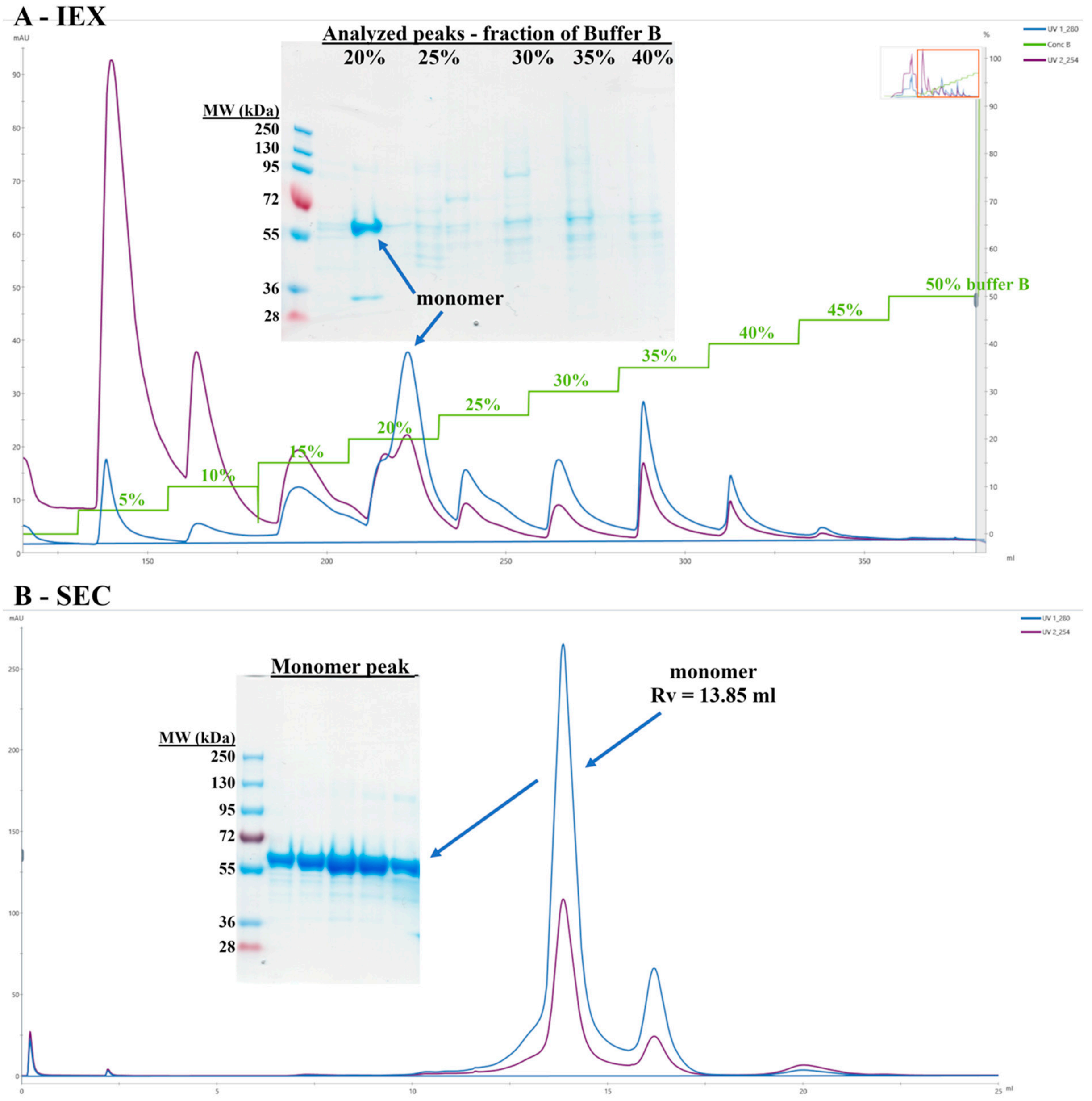

Figure S2. IEX and SEC chromatography of the Exo protein. **A**. IEX chromatogram with the recorded absorbance (254 nm - purple and 280 nm - blue) and conductivity (green) traces presents initial separation of the protein dialysate. The peak fractions were resolved by reducing SDS-PAGE, revealing electrophoretic mobility of the monomer and lack of either dimeric or oligomeric forms. **B.** SEC chromatogram of the separated IEX monomeric fractions (254 nm - purple and 280 nm - blue) and their subsequent analysis by reducing SDS-PAGE.

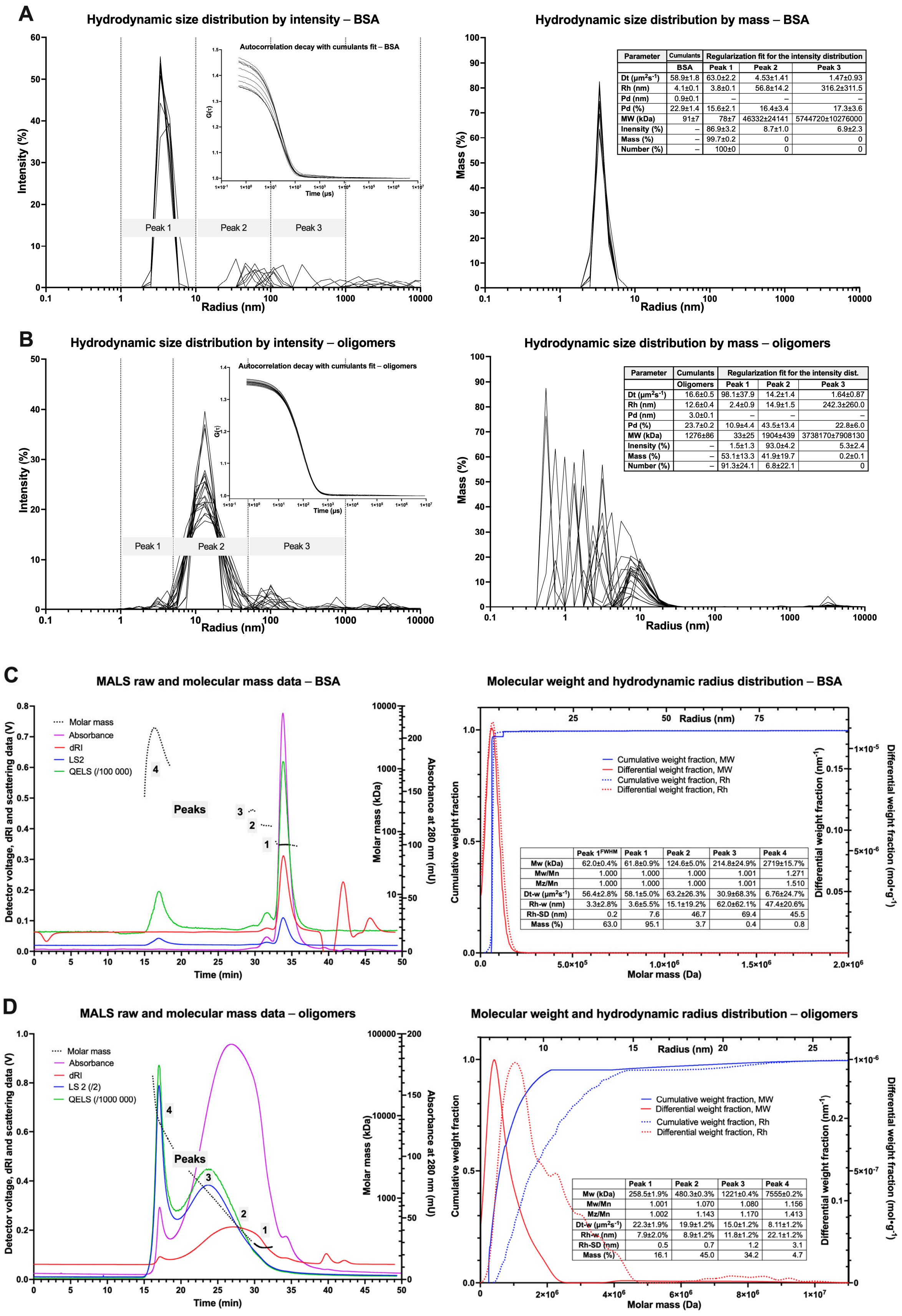

Figure S3. Results of DLS and MALS measurements for BSA and oligomeric fractions of DT390-IL-13. **A**. DLS of BSA. **B**. DLS of the oligomers. **C**. MALS of BSA. **D**. MALS of oligomers.

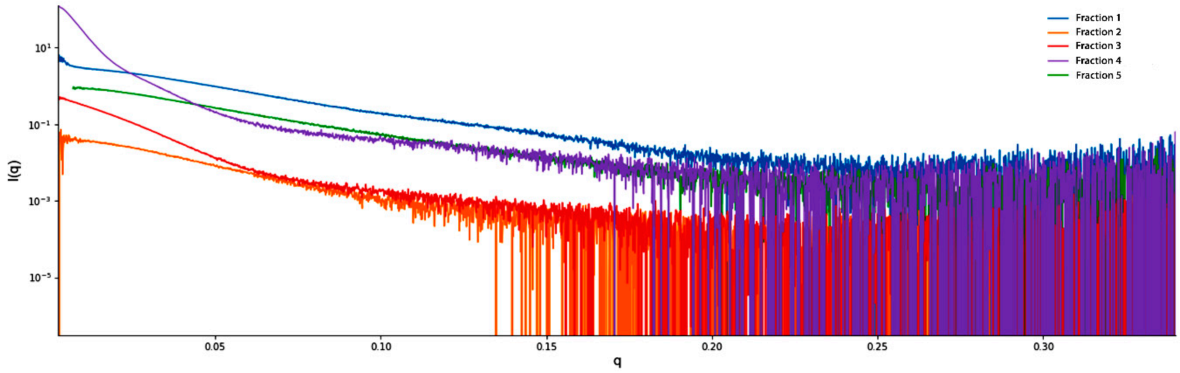

Figure S4. SAXS scattering profiles for all SEC-FPLC fractions.

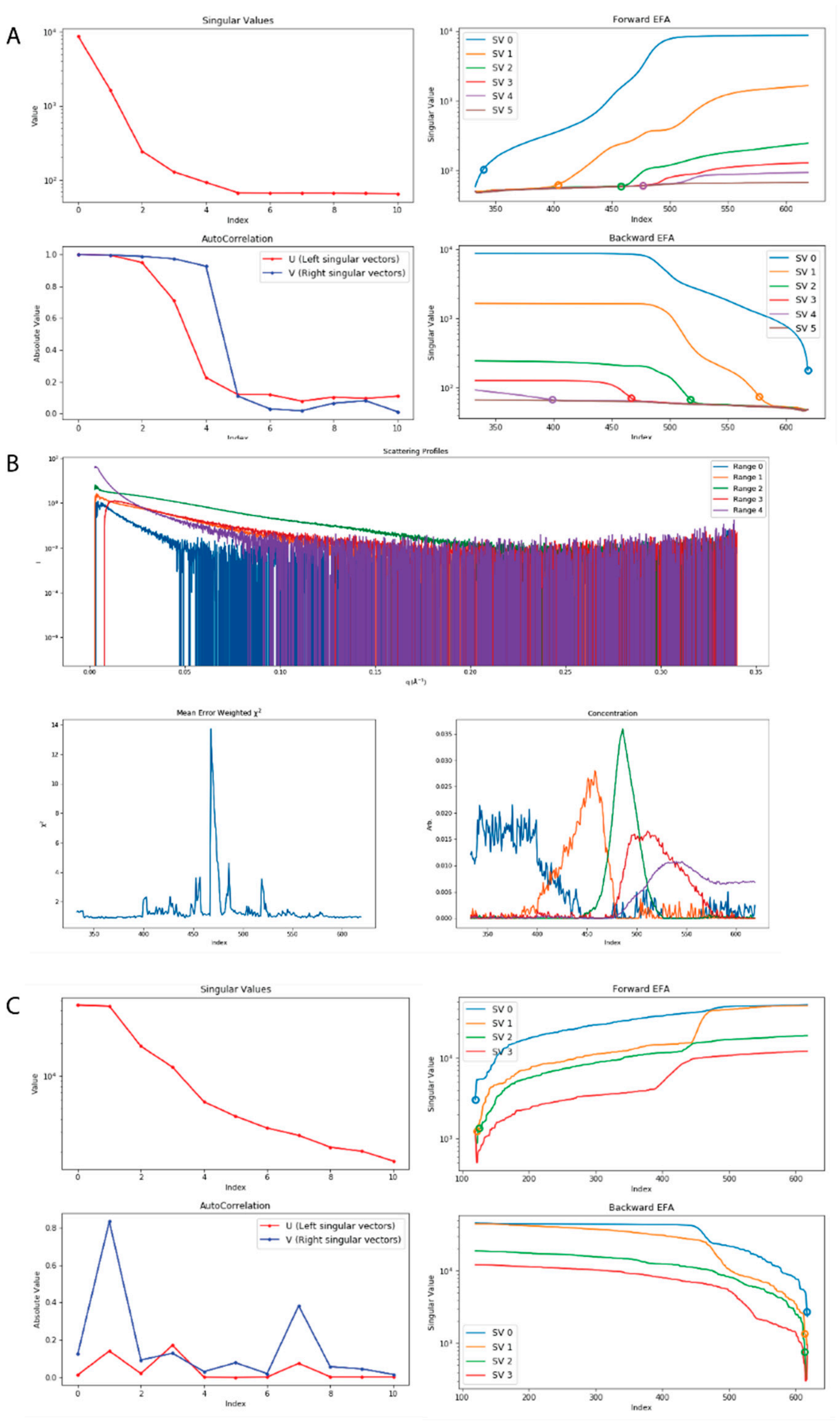

Figure S5. Exemplary SVD-EFA results. **A.** Successful EFA decomposition for fraction **1** resulting in a number of datasets representing distinctive scattering species in the solution. **B.** Scattering profiles for each decomposed datasets (up) and their respective time windows. **C.** Unsuccessful SVD-EFA decomposition for fraction **2**.

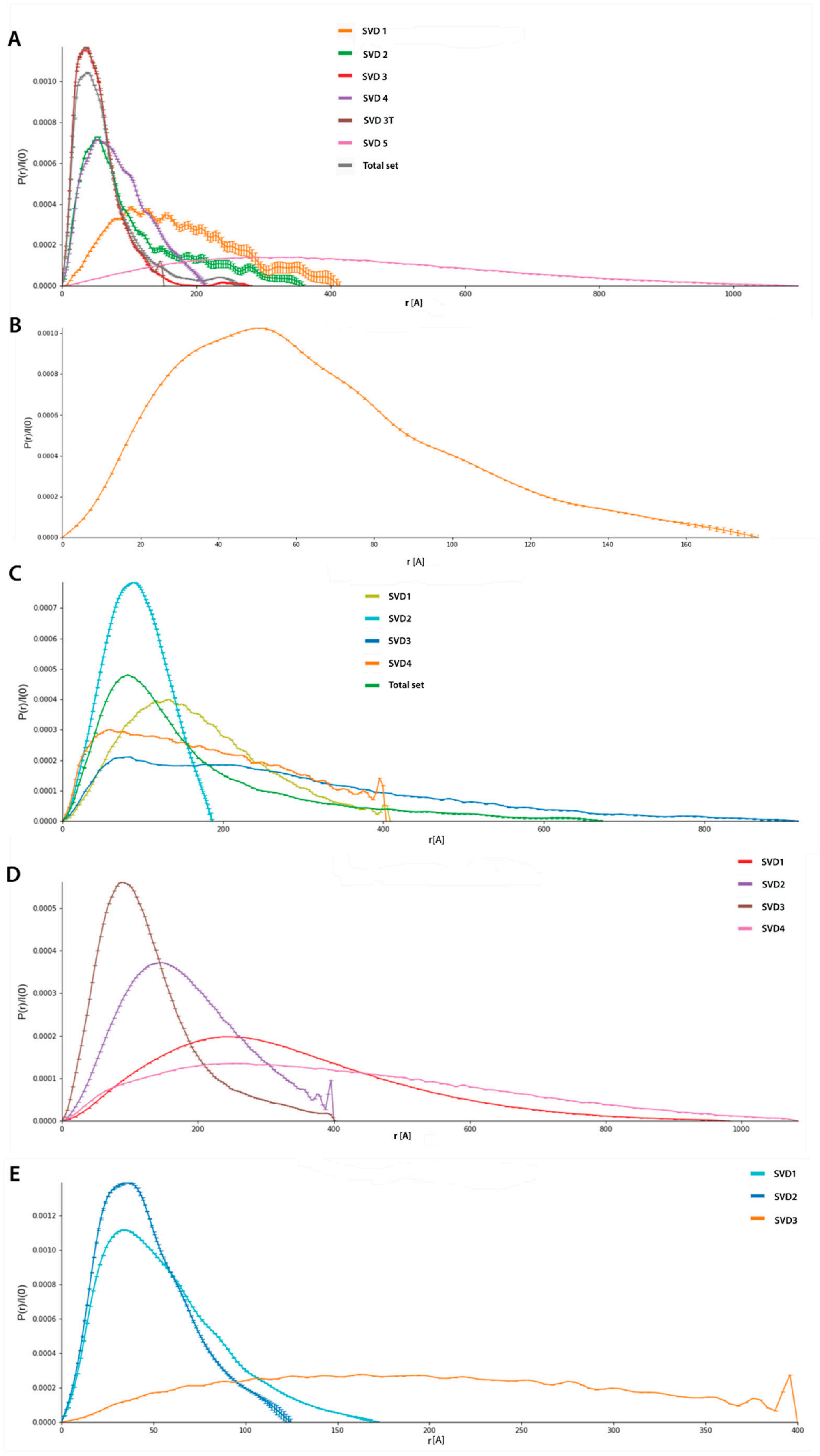

Figure S6. *P*(r) functions for all SVDs. Panels **A**-**E** correspond to fraction **1**-**5,** respectively.

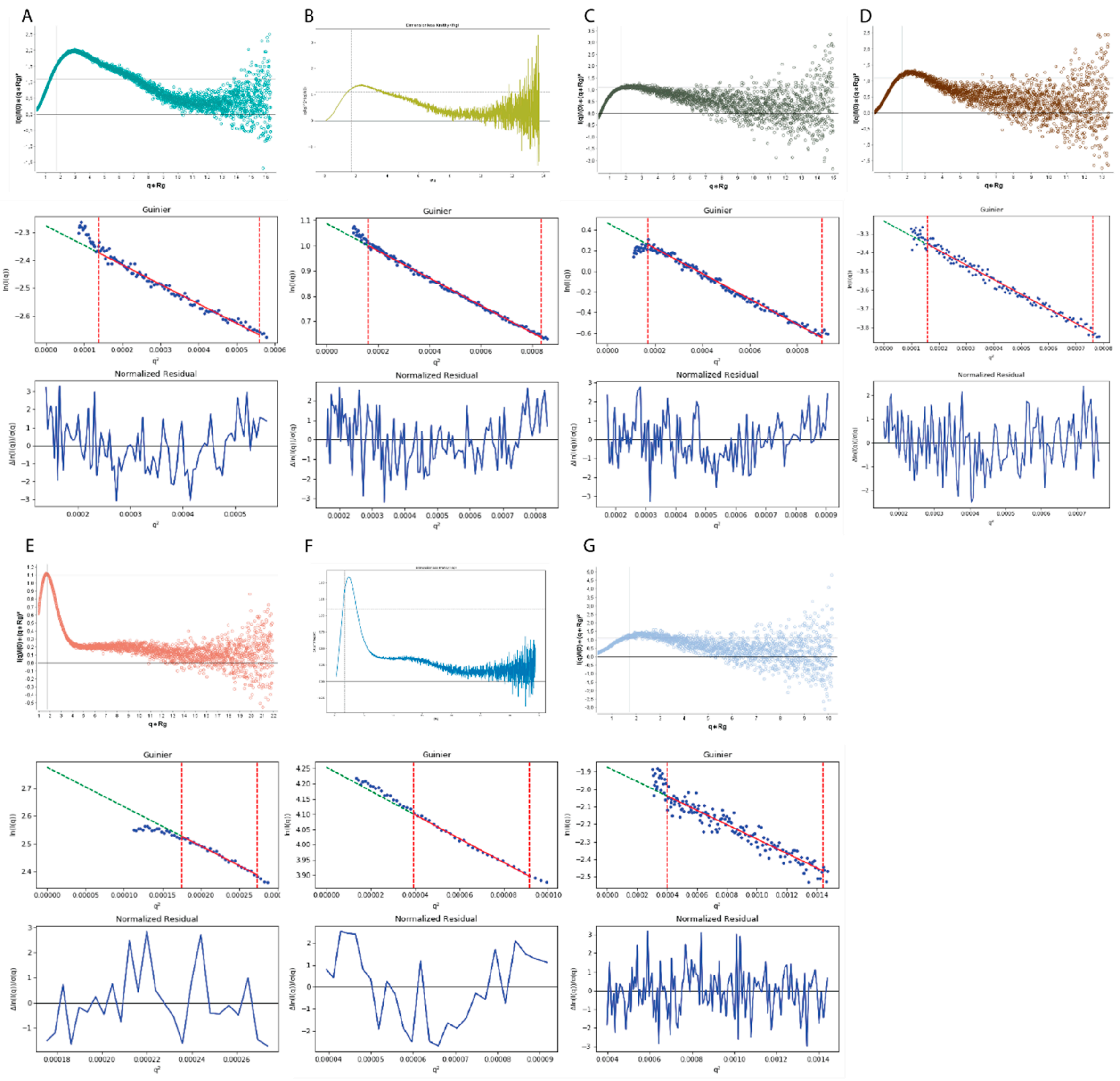

Figure S7. Guinier and dimensionless Kratky plots for selected datasets. For each panel the composition is as follows: Kratky plot (up), Guinier plot (middle), normalized residuals for Guinier fitting (down). Datasets: **1.3** (**A**), **1.3T** (**B**), **1.4** (**C**), **2.0** (**D**), **3.2** (**E**), **4.3** (**F**), **5.1** (**G**). A maximum located near the $R_{g}q$ = 1.1 observed for **3.2** and **4.3** is a further indicator of compactness.

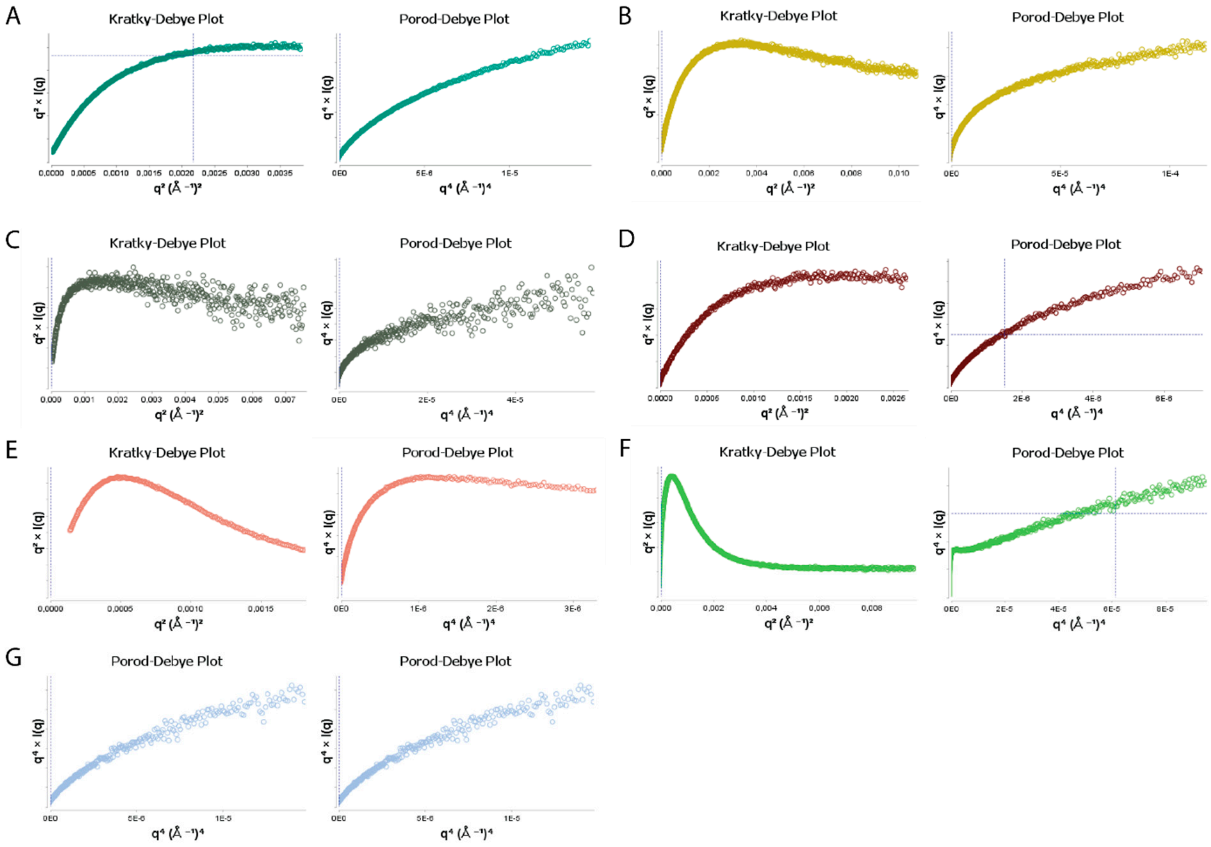

Figure S8. Flexibility analysis of selected datasets. Datasets: **1.3** (**A**), **1.3T** (**B**), **1.4** (**C**), **2.0** (**D**), **3.2** (**E**), **4.3** (**F**), **5.1** (**G**).

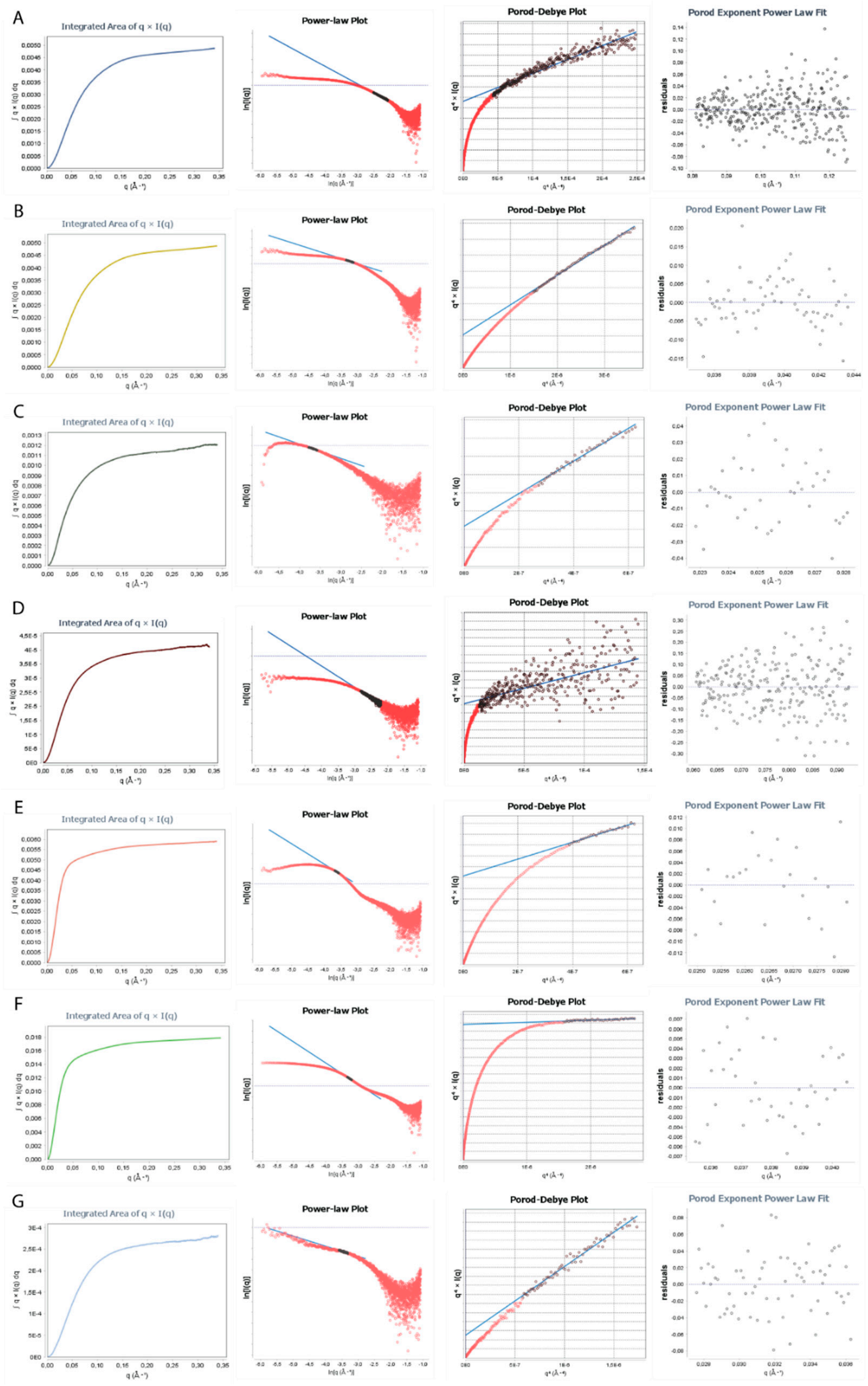

Figure S9. Volumetric analysis for selected datasets. For each panel the composition is as follows (from left to right): determination of V_c from integration of the whole scattering intensity as a function of q, fitting of truncated dataset to Porod's law to determine Porod exponent and correction factor to Porod invariant, distribution of residuals from the fitting. Datasets: **1.3** (**A**), **1.3T** (**B**), **1.4** (**C**), **2.0** (**D**), **3.2** (**E**), **4.3** (**F**), **5.1** (**G**).

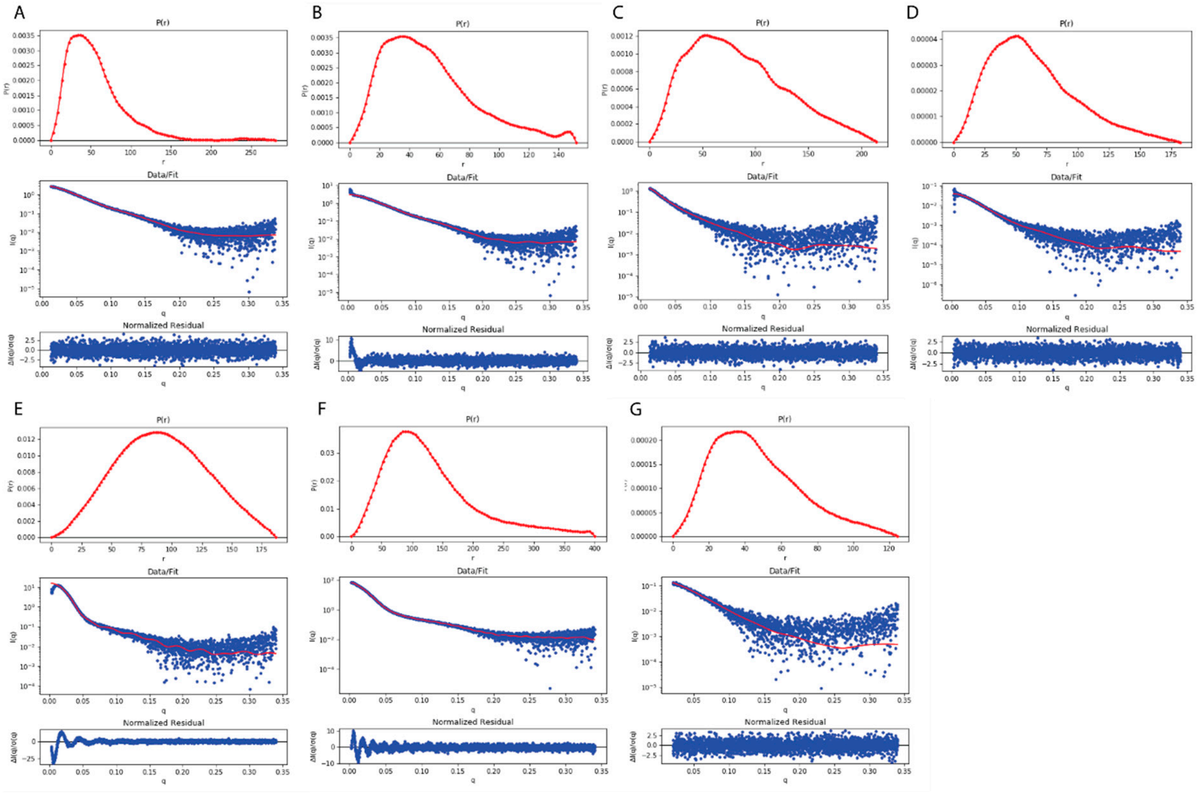

Figure S10. $P(r)$ functions and the errors of BIFT for selected datasets. For each panel the composition is as follows (from top to bottom): $P(r)$ function, fitting of $P(r)$ to experimental data, distribution of normalized residuals for the fitting. Datasets: **1.3** (**A**), **1.3T** (**B**), **1.4** (**C**), **2.0** (**D**), **3.2** (**E**), **4.3** (**F**), **5.1** (**G**).

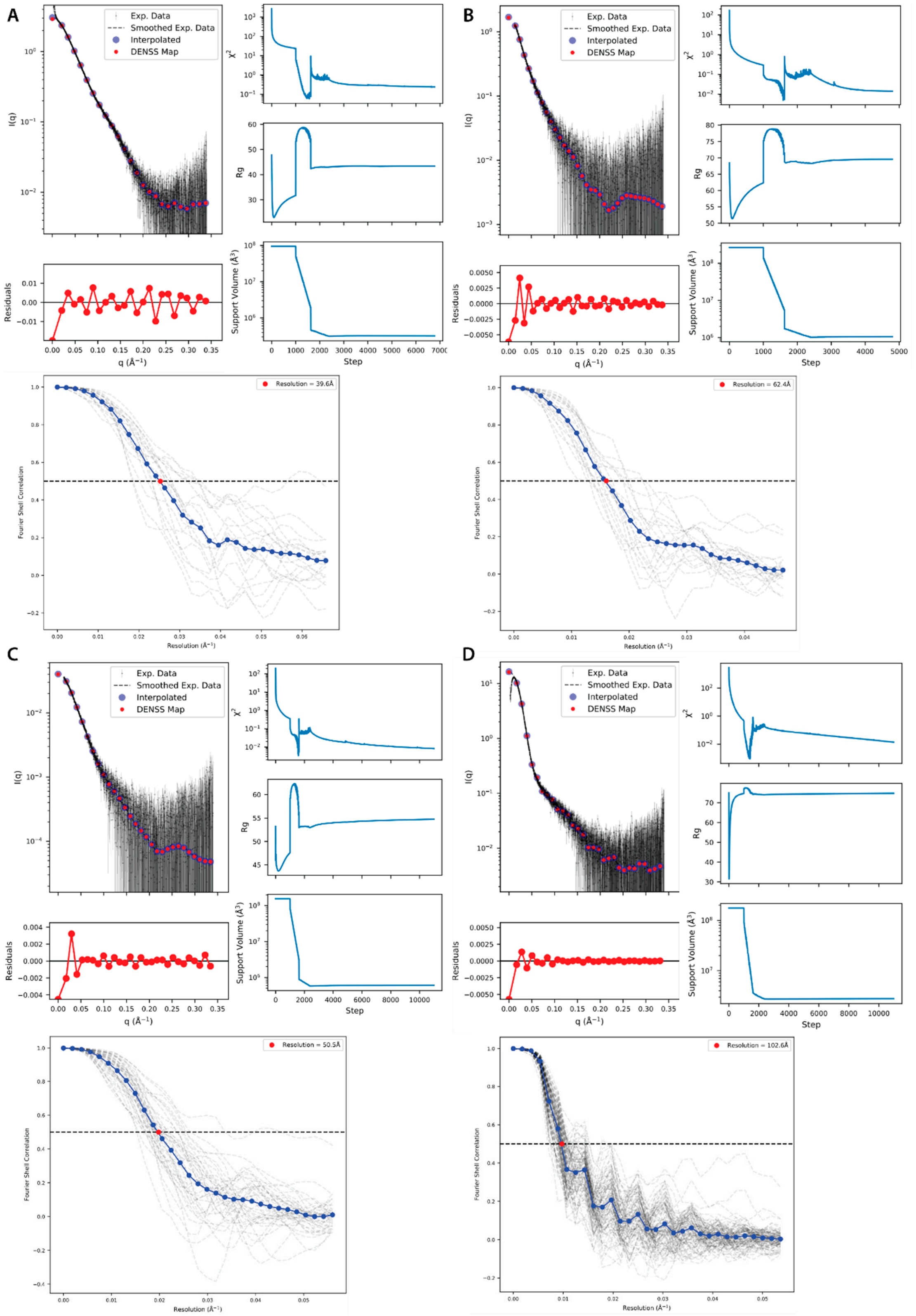

Figure S11. Quality assessment of ED reconstructions. For each panel the composition is as follows: fitting of theoretical $I(q)$ profiles from ED models to experimental $I(q)$ (left), χ^2 error, R_g and supported volume for each step of ED reconstruction, (right), averaged FSC as a function of reciprocal ED resolution. Datasets: **1.3** (**A**), **1.3T** (**B**), **1.4** (**C**), **2.0** (**D**).

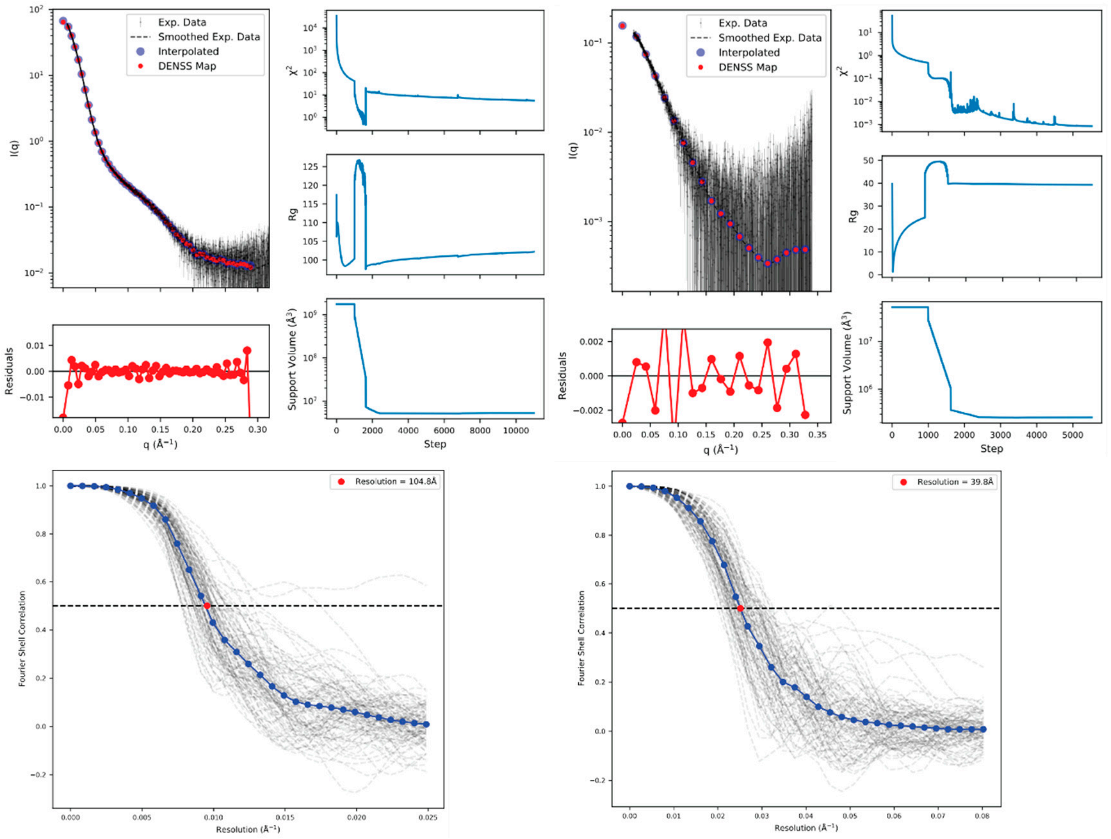

Figure S12. Quality assessment of ED reconstructions. For each panel the composition is as follows: fitting of theoretical $I(q)$ profiles from ED models to experimental $I(q)$ (left), χ^2 error, R_g and supported volume for each step of ED reconstruction, (right), averaged FSC as a function of reciprocal ED resolution. Datasets: **3.2** (**E**), **4.3** (**F**), **5.1** (**G**).

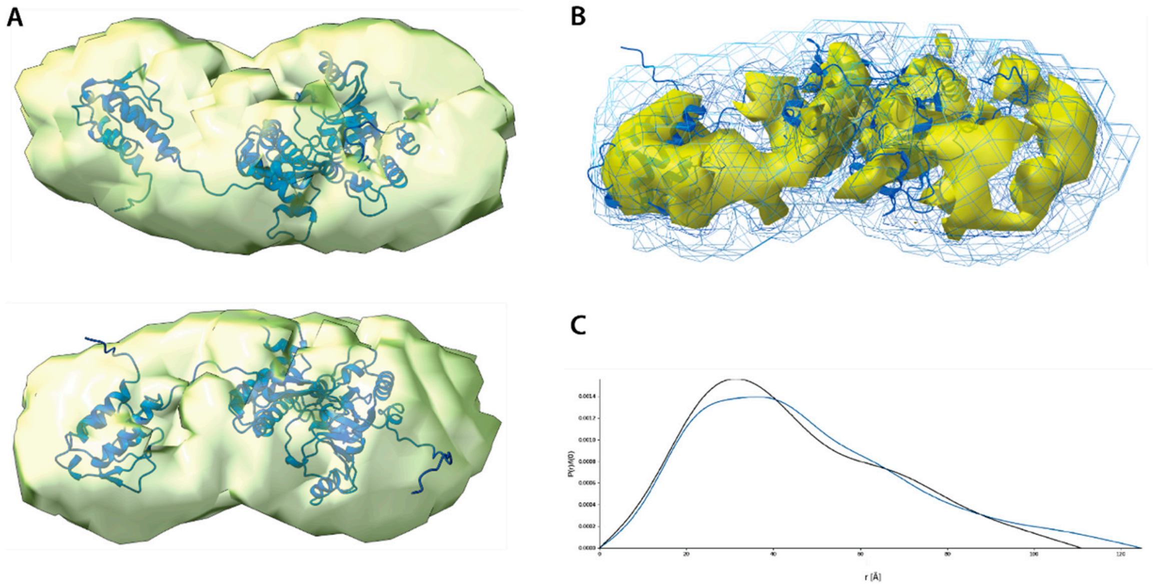

Figure S13. SAXS structural modelling of Exo-construct. **A.** Alignment of atomic model onto ED reconstruction from both sides. **B.** Layers of different electron density showing good coverage of polypeptide chain. **C.** Comparison of $P(r)$ function of atomic model (blue) to experimental $P(r)$ function (black).

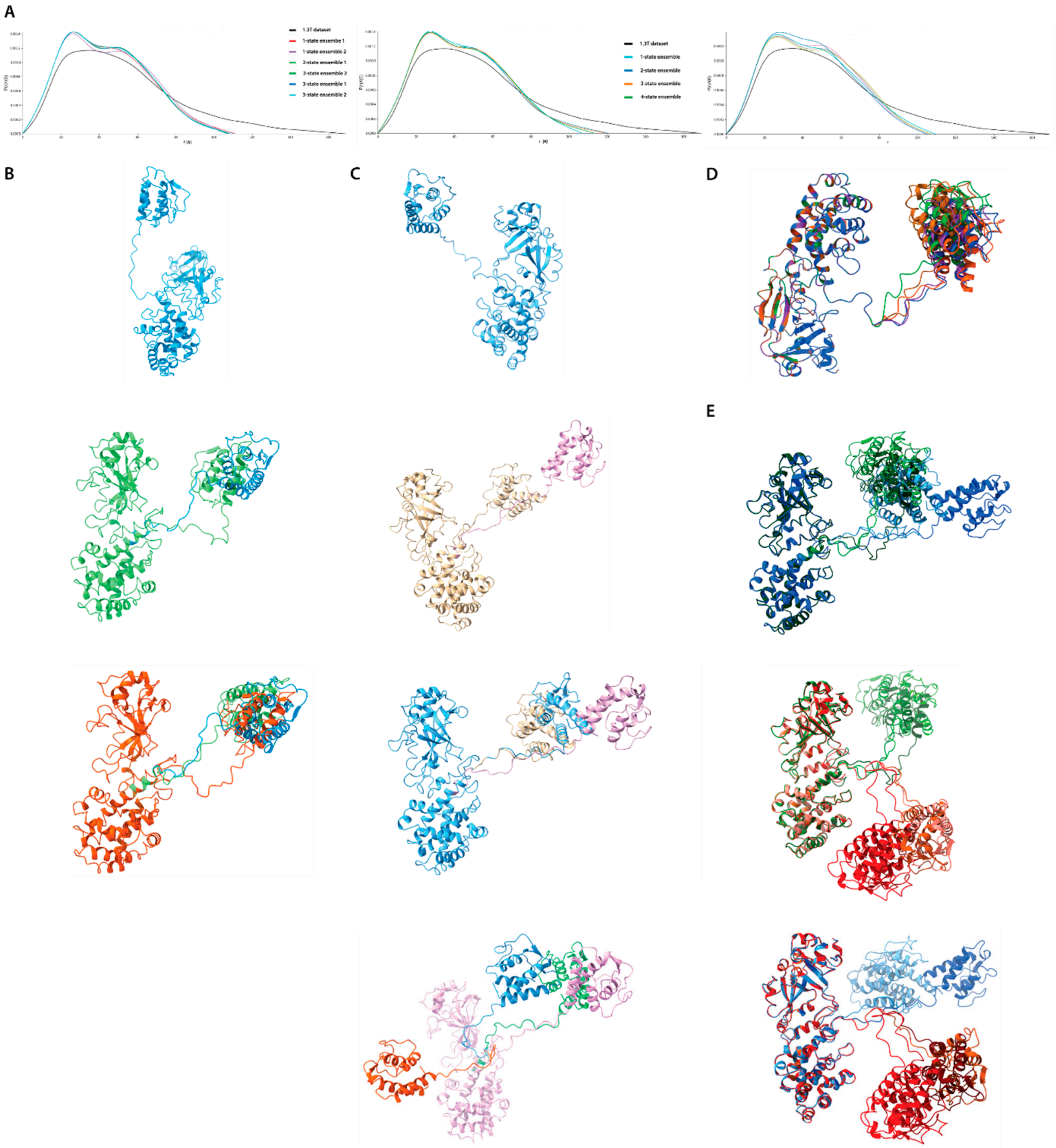

Figure S14. Ensemble analysis for DT protein. **A**. *P*(*r*) function of statistical ensembles and set of conformers generated by different methods compared to native dataset. BILBOMD (left), MultiFOXS (center) and SASRef (right). **B** 1-, 2-, and 3-states ensembles from BILBOMD. **C**. 1-, 2-, 3- and 4-states ensembles from MultiFOXS. **D.** Set of conformers from SAXRef. **E.** Structural alignments between ensembles generated by different methods BILBOMB, MultiFOXS and SAXRef, different conformers in each ensemble are depicted using palettes of following colours: green, blue and red, respectively.

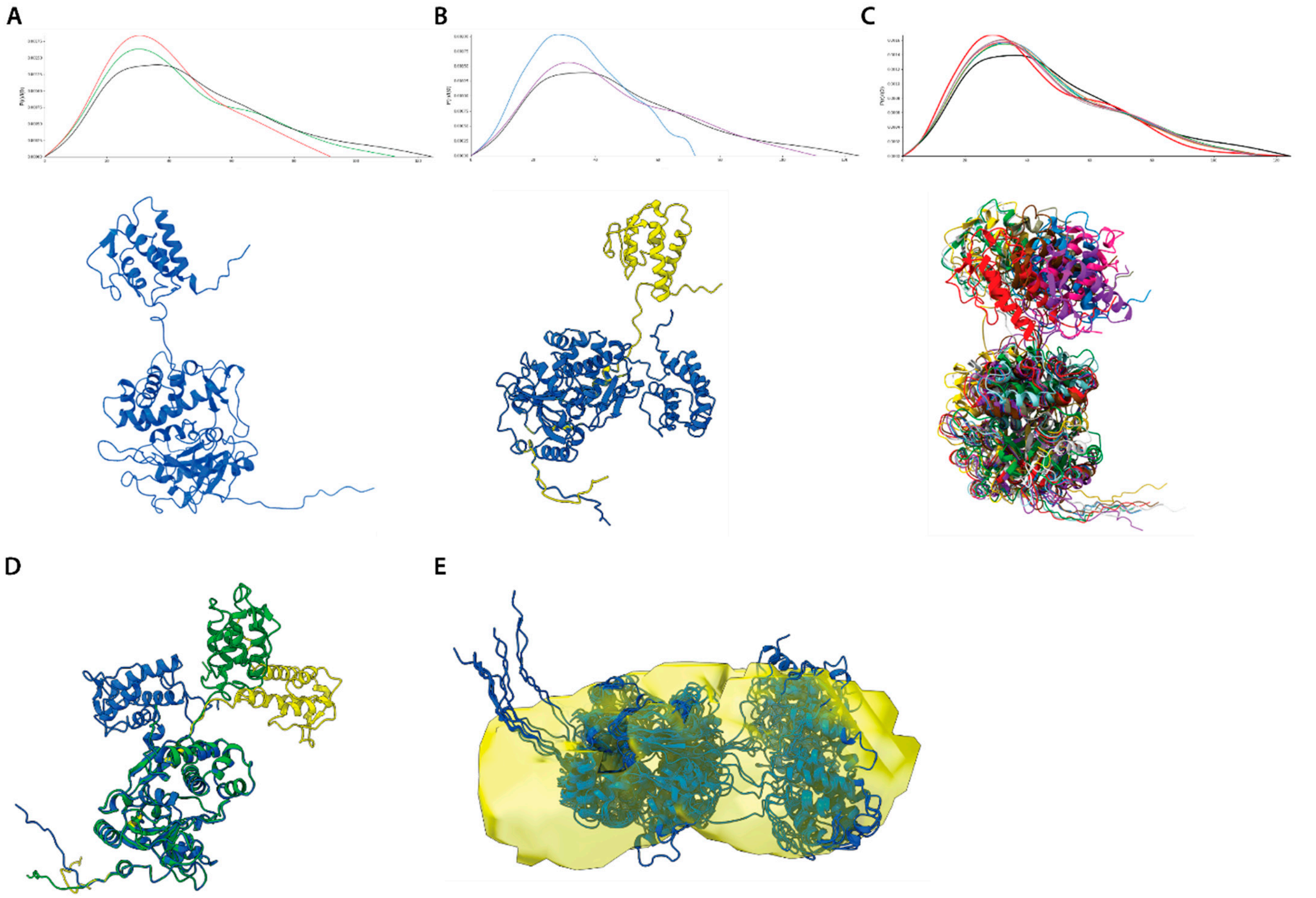

Figure S15. Ensemble analysis for Exo protein. *P*(*r*) function of statistical ensembles and set of conformers generated by different methods compared to native dataset. BILBOMD (**A**), MultiFOXS (**B**) and SASRef (**C**). (**D**) 1-, 2-, and 3-states ensembles from BILBOMD. **C**. 1-, 2-, 3- and 4-states ensembles from MultiFOXS. **D.** Structural alignments between ensembles generated by different methods BILBOMB, MultiFOXS and SAXRef, different conformers in each ensemble are depicted using palettes of following colors: green, blue and red respectively. (**E**). Conformational ensemble from SASRef fitted to ED model

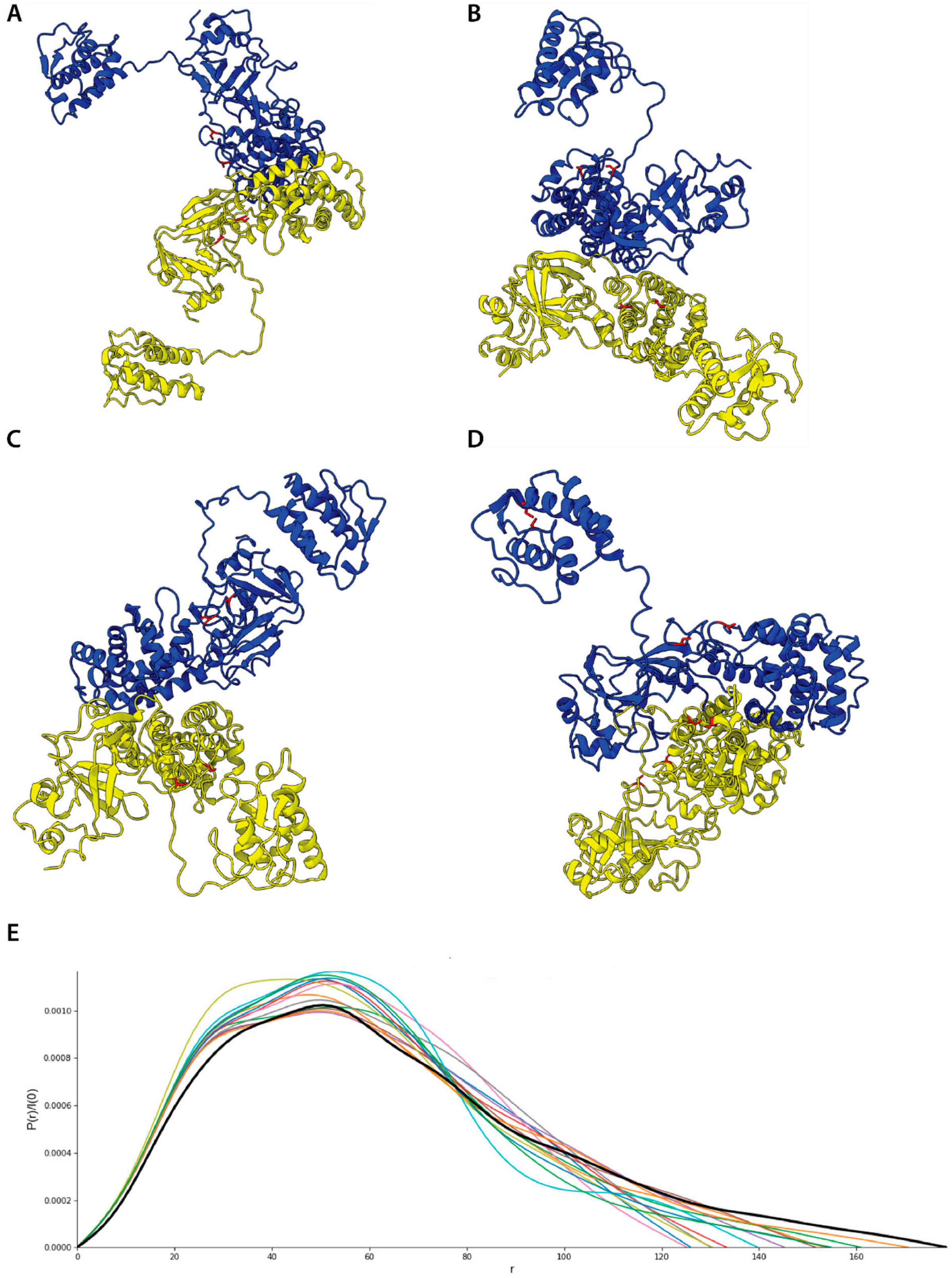

Figure S16. FoXSDock rigid docking. (**A**–**D**) structure of selected dimers composed of following conformers calculated by MultiFOXS: *e1-1*, *e1-1* (**A**); *e2-1*, *e2-2* (**B**); *e3-2*, *e3-2* (**C**); *e4-2*, *e4-3* (**D**). (**E**). *P*(*r*) functions computed for a set of dimers compared to experimental *P*(*r*) function for **2.0** dataset corresponding to dimer.

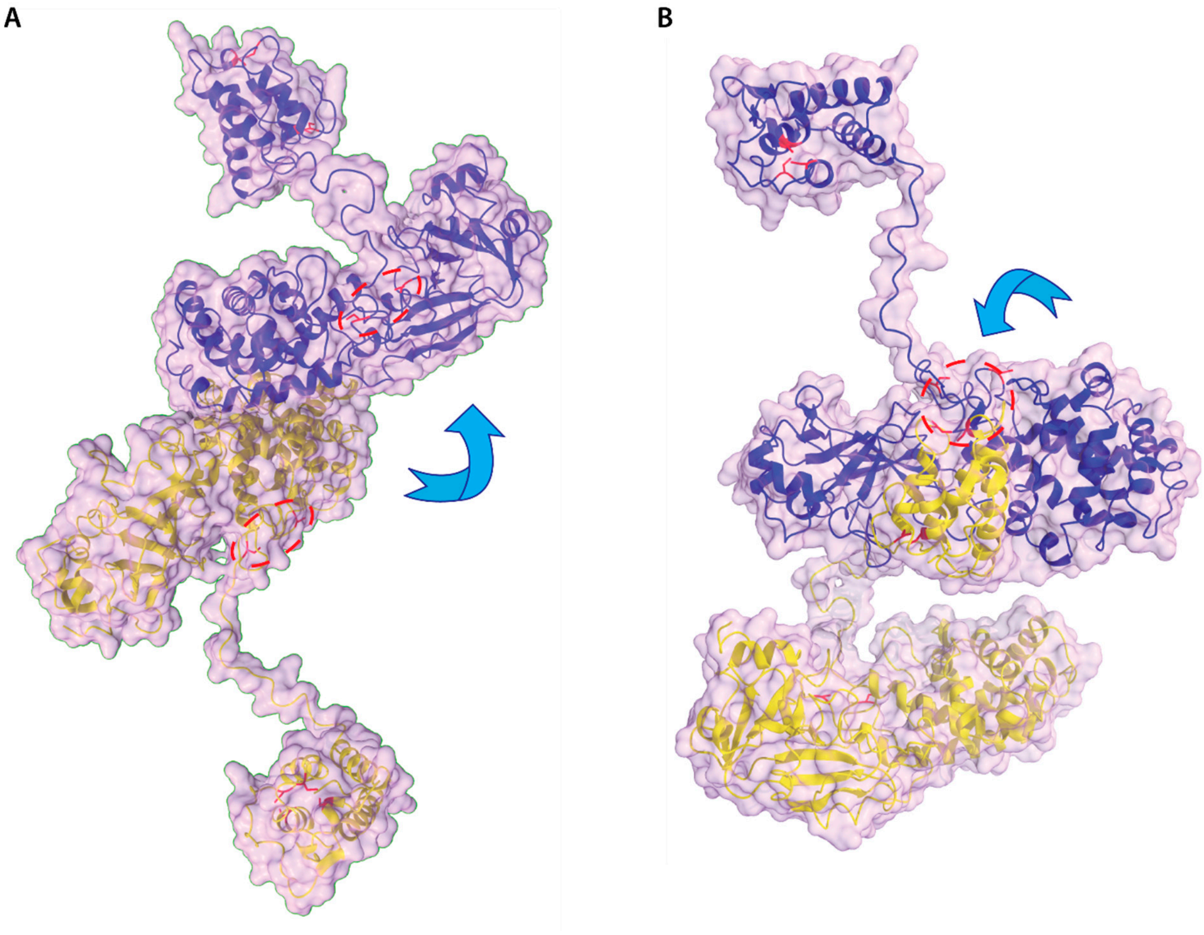

Figure S17. Approximate models of endo-dimer (**A**) and exo-dimer (**B**). Arrows indicate suggested conformational changes necessary to form disulfide bridges between molecules.

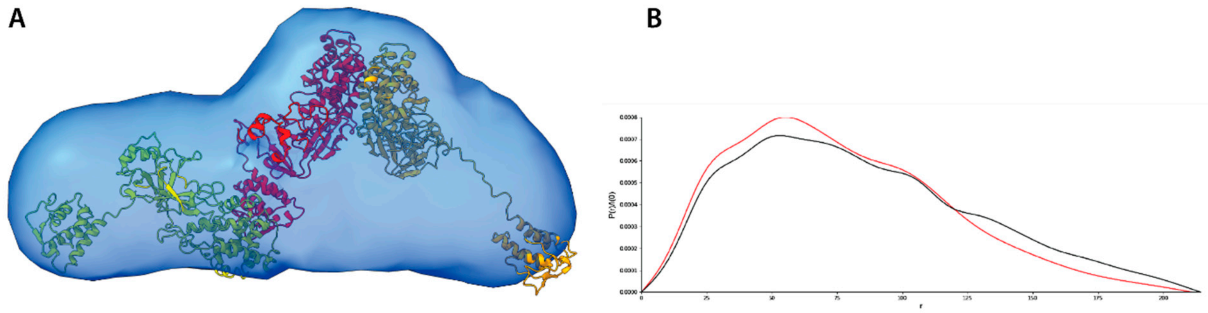

Figure S18. Estimate structure of the proposed trimer. **A.** Trimeric structure fitted to ED model **B.** Comparison of $P(r)$ functions of native datasets used for modelling (black) with the $P(r)$ function obtained from the trimer.

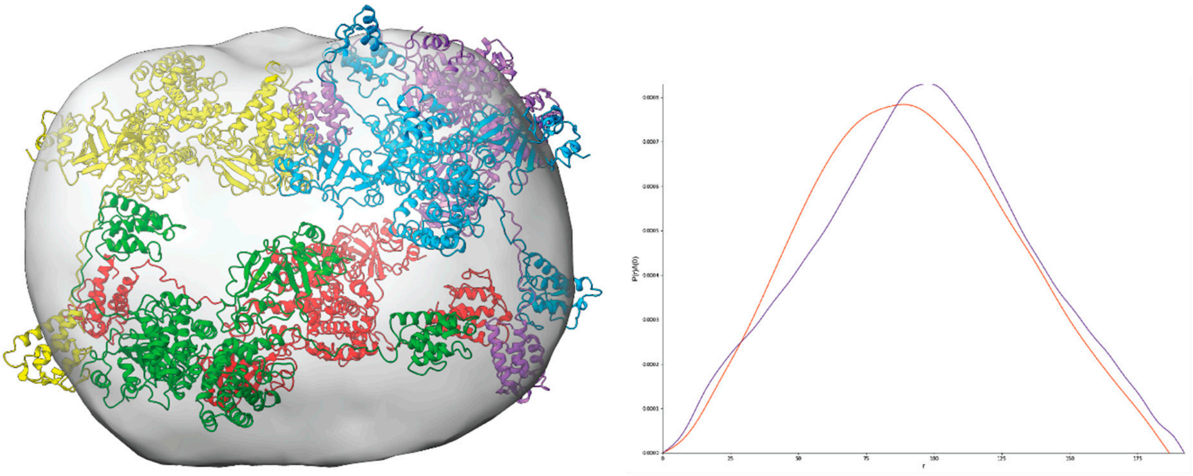

(**A**) (**B**)

Figure S19. Proposed structure of 10-mer based on SAXS data. **A**. Fitting of the estimated model to ED reconstruction. **B**. Comparison of experimental *P*(*r*) function of 3.2 dataset (orange) to theoretical *P*(*r*) calculated for the 10-mer.

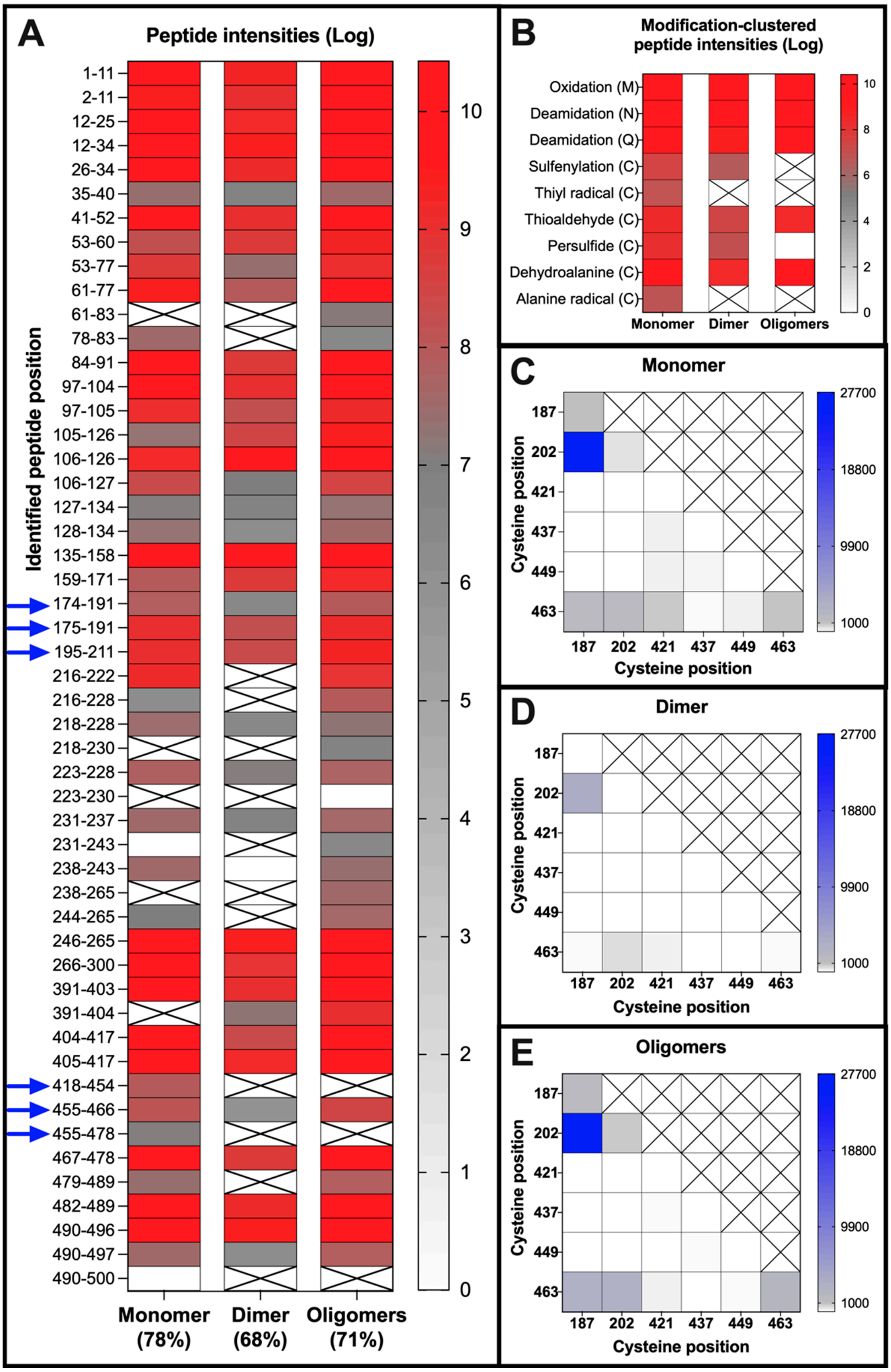

Figure S20. MS analysis of DT390-IL-13 protein. (**A**). Sequence coverage, blue arrows indicate peptides containing disulfide bonds. (**B**). Analysis of post-translational modifications for each fraction. Gray-red gradient indicates intensities for a given modification. (**C**-**E**) mapping of disulfide bonds for monomeric (**C**), dimeric (**D**) and oligomeric (**E**) fractions. Gray-blue gradient indicates Andromeda Score for particular disulfide bond.

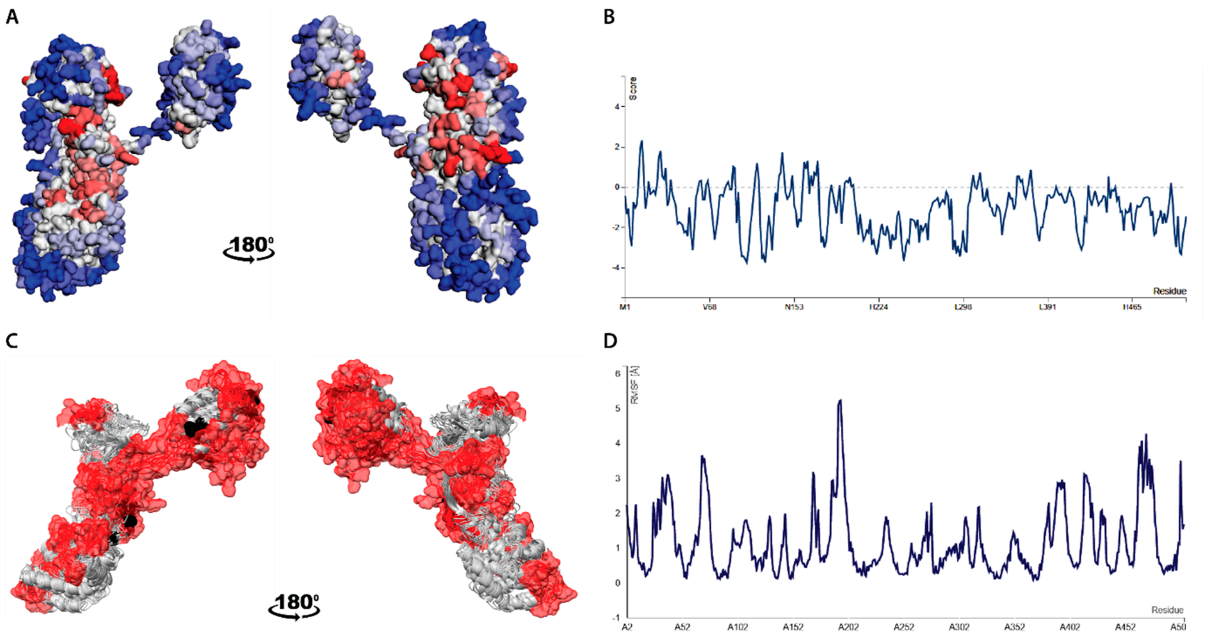

Figure S21. Computational analysis of flexibility and propensity for aggregation of DT390-IL-13. **A**. Mapping of aggregation score on molecular surface using BWR coloring scheme, where low aggregation score values are blue and high are red. **B**. Plot of aggregation score for each residue. **C**. Flexible residues (depicted as red) mapped onto molecular surface of computed conformational ensemble. Cysteine residues are depicted in black. **D**. Plot of flexibility score for each residue.

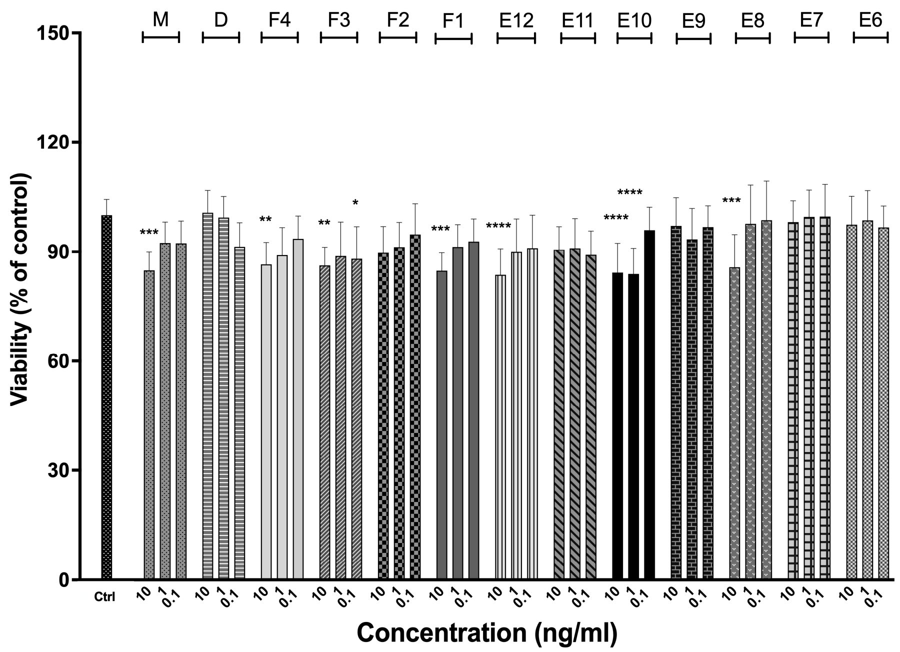

Figure S22. The viability of LN229 glioblastoma cell line in presence of the cytotoxin monomer (M), dimer (D), and oligomers (F4-E6), measured by the MTS assay, in three concentrations, 0.1, 1.0, and 10.0 ng/ml (which correspond to 1.8, 18.1, and 181.3 pM concentrations).
